# Supplementary material for: Atypical cortical feedback underlies failure to process contextual information in the superior colliculus of Scn2a+/− autism model mice
Source: Nat Commun. 2025 Sep 30;16:8659. doi: 10.1038/s41467-025-63788-2 (PMC12484672; doi:10.1038/s41467-025-63788-2)
Supplement: Supplementary file 1 — Supplementary Information File [file 41467_2025_63788_MOESM1_ESM.pdf]

Atypical cortical feedback underlies failure to process contextual information in the superior colliculus of Scn2a$^{+/-}$ autism model mice

Leiron Ferrarese, Hiroki Asari

Supplementary Information

- Supplementary Figure 1
- Supplementary Figure 2
- Supplementary Figure 3
- Supplementary Figure 4
- Supplementary Figure 5
- Supplementary Figure 6
- Supplementary Table 1
- Supplementary Table 2

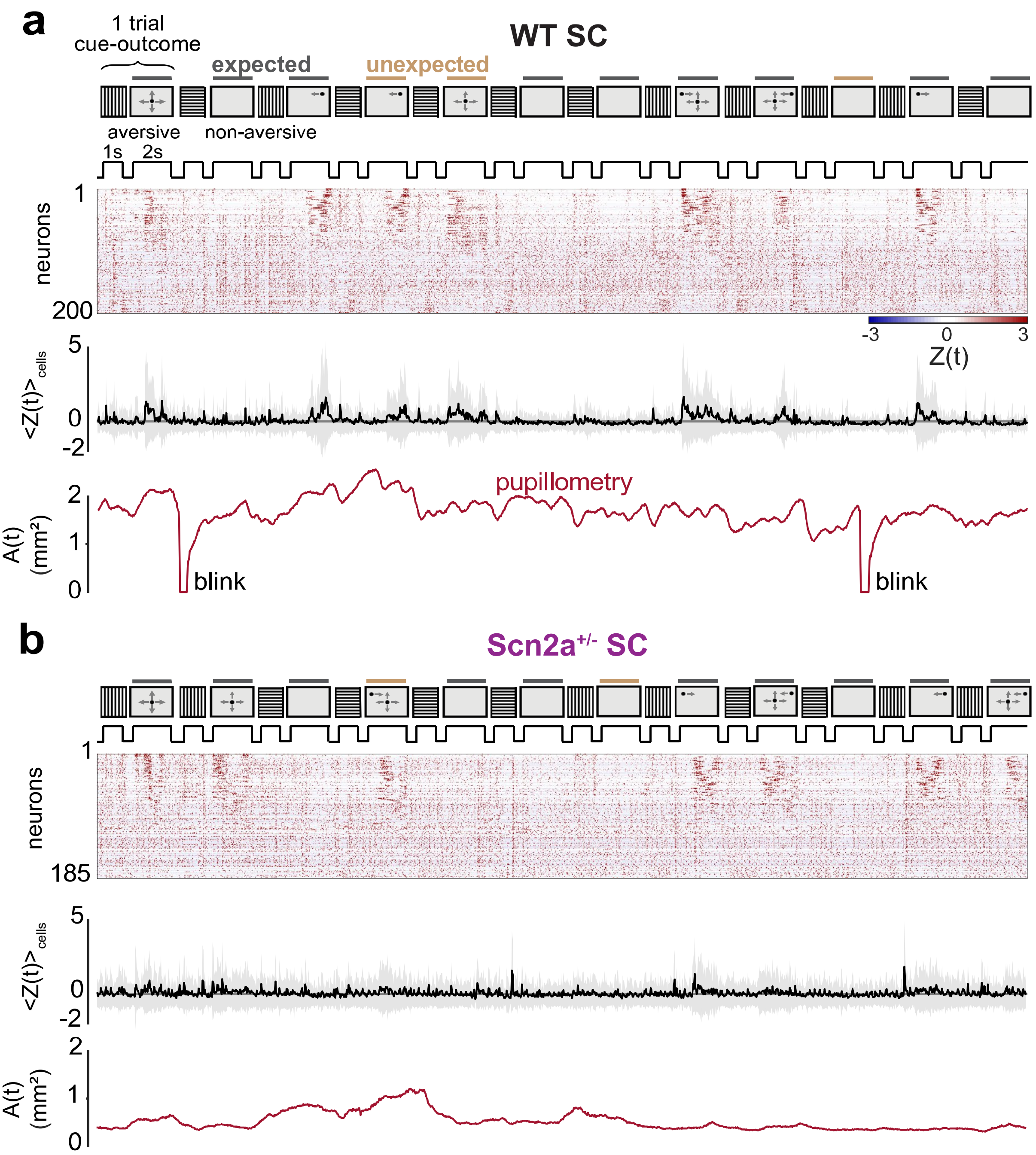

Supplementary Figure 1: Representative neuronal activity of the mouse superior colliculus and pupil dynamics in response to the cue-outcome stimulus sequences.
a Trial-by-trial response example obtained from a wild-type (WT) mouse during the early stable (ES) epoch, with 83% expected and 17% unexpected outcomes. *From top to bottom*: The normalized spiking activity of the superior colliculus (SC) neurons $Z(t)$ (z-score), estimated from recorded calcium dynamics; corresponding population average response $\langle Z(t) \rangle_{\text{cells}}$ (gray shade, ±1 standard deviation); and concurrent dynamics of the estimated pupil size $A(t)$ in mm^2. **b** Corresponding data from a representative Scn2a$^{+/-}$ mouse.

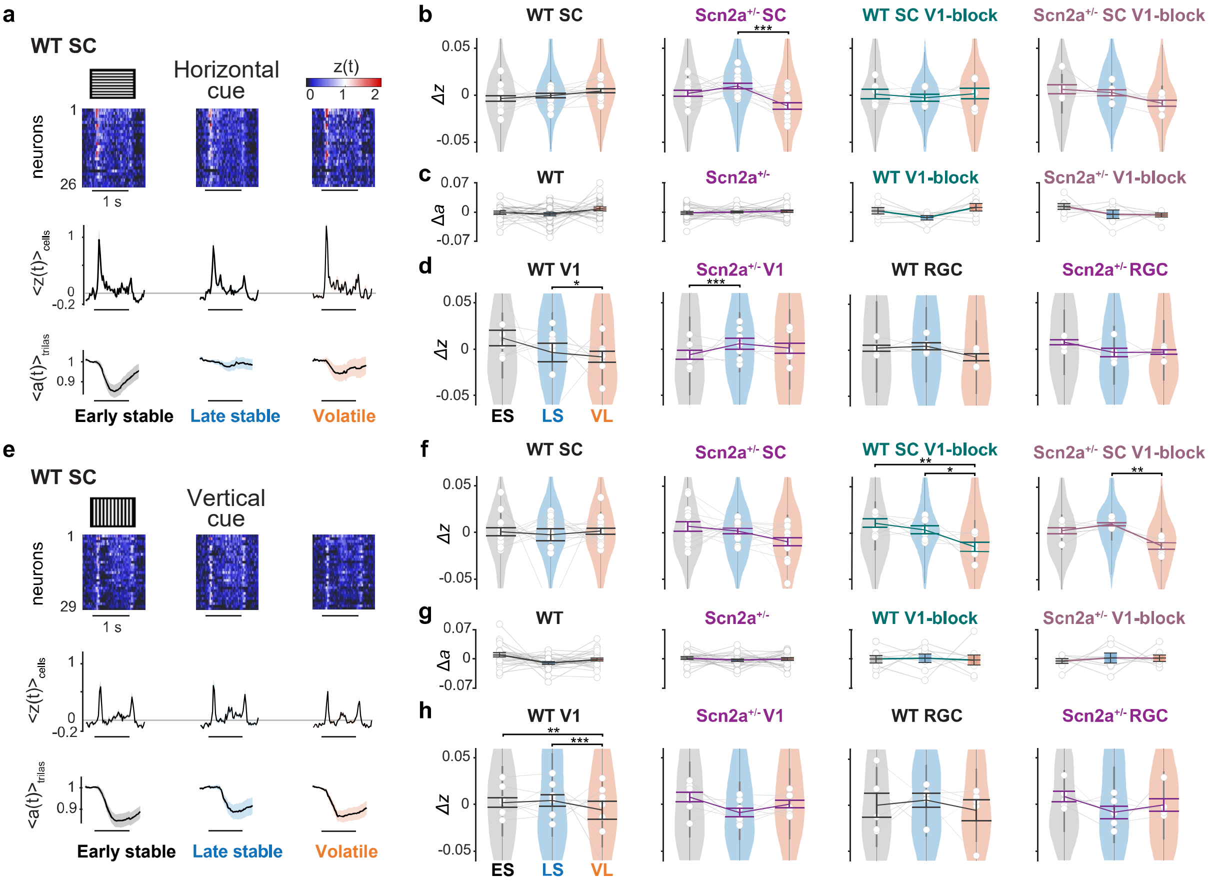

Supplementary Figure 2: Contextual modulation patterns of the cue stimulus responses in the mouse early visual system.

Here and thereafter, ***, p<0.001; **, p<0.01; *, p<0.05 with Tukey-Kramer test for pairwise comparisons (see Supplementary Table 1 and Methods for details). **a** Heatmap ($z(t)$; sorted with $\max[z(t)]$; top) and the population average ($\langle z(t)\rangle_{\text{cells}}$; shaded area, standard error of the mean (s.e.m.); middle) of the responses to one of the cue stimuli (horizontal gratings) in each epoch for SC neurons of a representative WT mouse. Corresponding pupil dynamics are shown at the bottom ($\langle A(t)\rangle_{\text{trials}}$, average proportional change in pupil area; shaded area, s.e.m.). **b** Distributions of the normalized horizontal grating responses (Δz) of SC cells in WT or Scn2a$^{+/-}$ mice, with or without chemogenetically blocking the projections from the primary visual cortex (V1), across epochs. A decrease of the population response strength was observed in the SC of Scn2a$^{+/-}$ mice in the volatile (VL) epoch. **c** Normalized change in pupil size (Δa) upon horizontal grating stimulus presentations (from left to right, 25 WT mice, 24 Scn2a$^{+/-}$ mice, 8 WT mice with V1-block, 7 Scn2a$^{+/-}$ mice with V1-block). No contextual modulation was observed across epochs. **d** Distributions of the normalized horizontal grating responses of V1 or retinal ganglion cell (RGC) axons in WT or Scn2a$^{+/-}$ mice. No modulation was found in RGC responses in both genotypes, while reduction of the response strength across epochs was observed for V1 axons. **e-h** corresponding figure panels in response to the other cue stimulus (vertical gratings). In WT mice, no modulation was observed in RGC or pupil dynamics, while V1 axons showed adaptation, consistent with the responses to the vertical grating stimuli. In Scn2a$^{+/-}$ mice, no contextual modulation was identified.

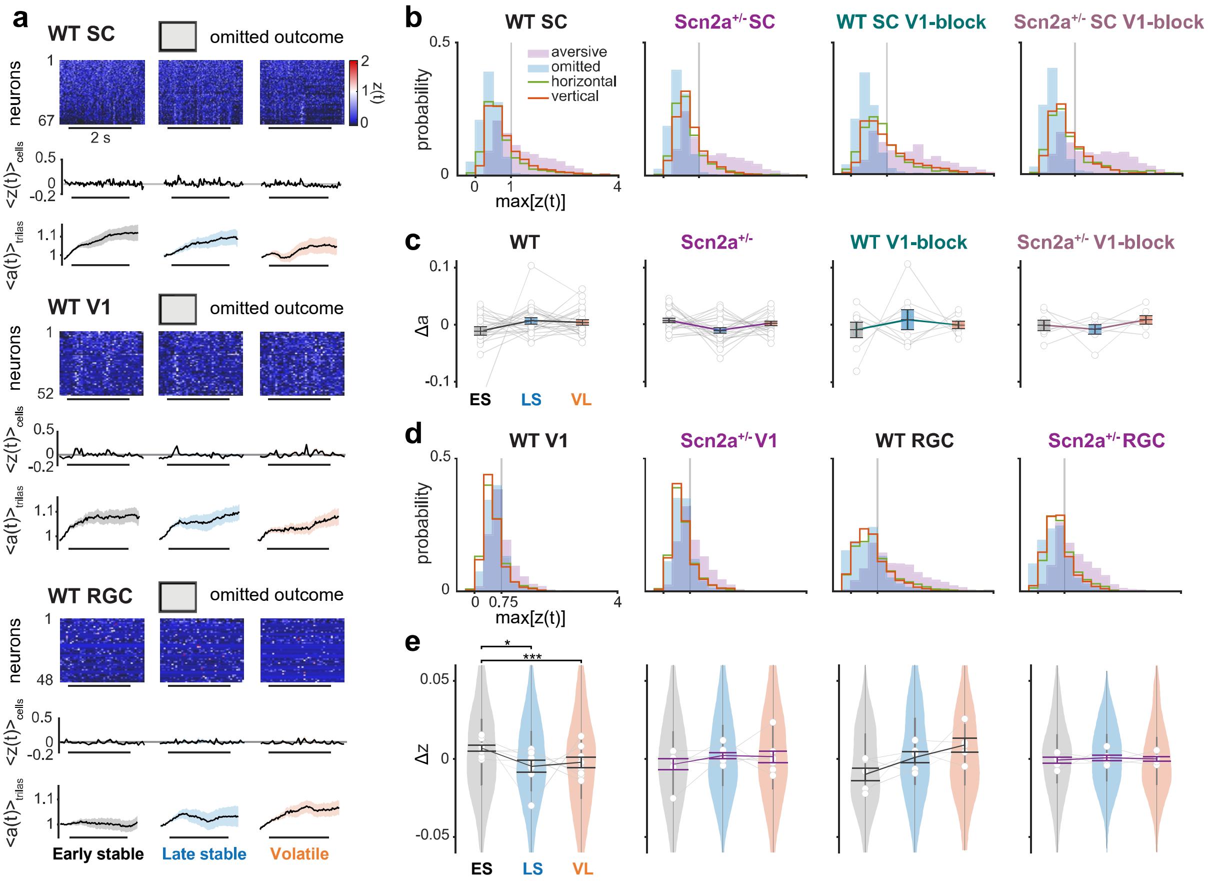

Supplementary Figure 3: no contextual modulation was observed in response to the omitted stimulus.

a Heatmap ($z(t)$; sorted with $\max[z(t)]$; top) and the population average ($\langle z(t)\rangle_{\text{cells}}$; shaded area, s.e.m.; middle) of the omitted stimulus responses in each epoch for SC neurons (top), V1 axons (middle), and RGC axons (bottom), each along with the pupil dynamics ($\langle\langle A(t)\rangle_{\text{trials}}$; shaded areas, s.e.m.) from the representative WT mice. **b** Probability distributions of SC responses ($\max[z(t)]$) to each stimulus type in WT or Scn2a$^{+/-}$ mice, with or without chemogenetically blocking V1 input. SC cells showed strong responses to the aversive stimuli (purple), modest responses to the cue stimuli (horizontal gratings, olive; vertical gratings, tangerine), and virtually no response to the non-aversive, omitted stimulus (blue) during the implicit visual learning paradigm. Hence, no comparison was made for the omitted response strength across epochs. **c** Normalized change in pupil size ($\Delta a = \bar{a} - \langle\bar{a}\rangle_{\text{epochs}}$) upon non-aversive omitted stimulus presentation in each epoch (from left to right: 25 WT mice, 24 Scn2a$^{+/-}$ mice, 8 WT mice with chemogenetic inhibition of V1-to-SC projections (V1-block), and 7 Scn2a$^{+/-}$ mice with V1-block): thin line, individual animal; error bars, population average and s.e.m.. **d** Probability distribution of V1 and RGC axonal responses to each stimulus type presented during the implicit learning task in WT and Scn2a$^{+/-}$ mice. Unlike SC cells, V1 and RGC axons demonstrated modest responses to the omitted stimulus. **e** Distributions of the normalized responses of V1 or RGC axons in WT or Scn2a$^{+/-}$ mice across epochs ($\Delta z = \bar{z} - \langle\bar{z}\rangle_{\text{epochs}}$). No contextual modulation was identified (Tukey-Kramer test for pairwise comparisons; see Supplementary Table 1 for details).

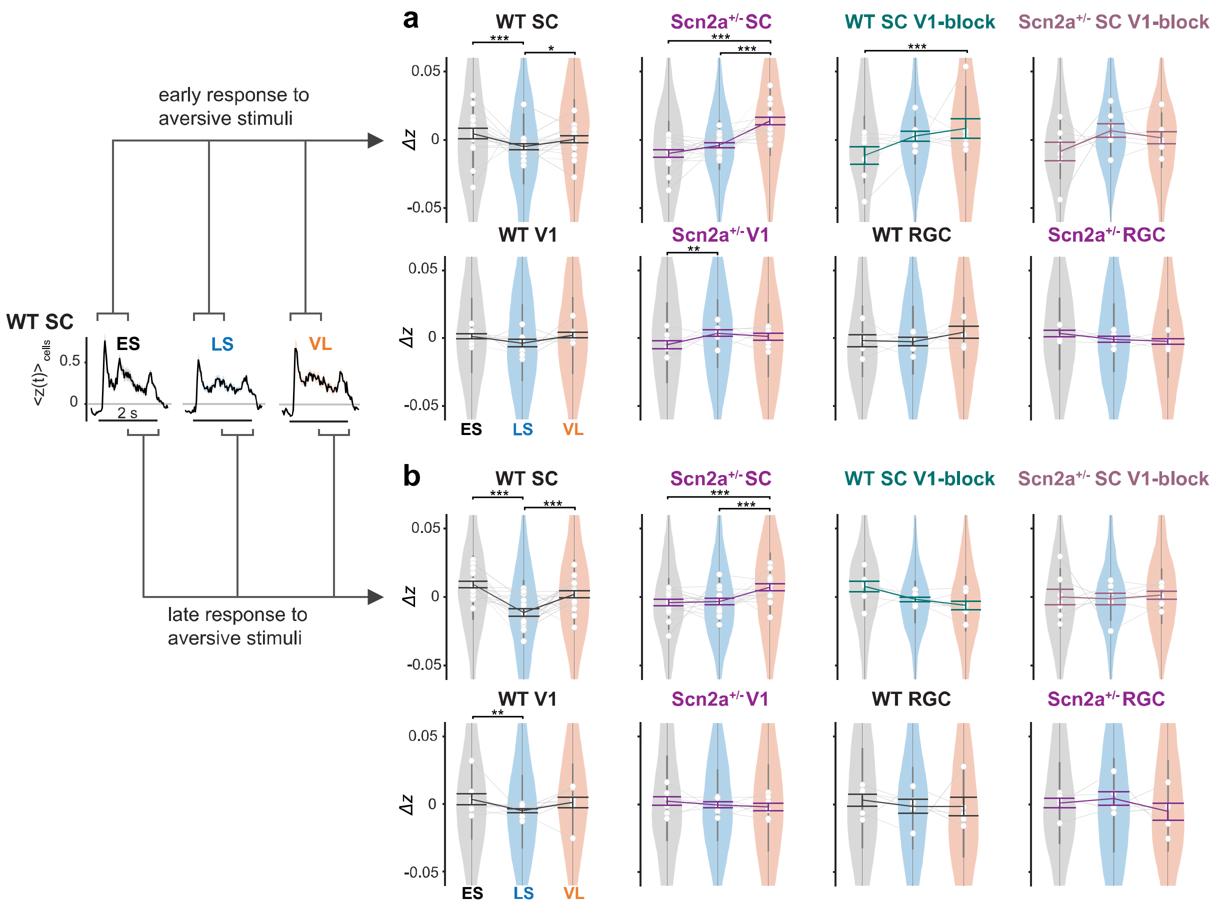

Supplementary Figure 4: Contextual-modulation was stronger for later response components in WT mice, but for earlier response components in Scn2a$^{+/-}$ mice.
We divided the aversive stimulus presentation period into the first (**a**; early responses) and second (**b**; late responses) halves in time (1 s each), and respectively analyzed the changes in the response strength across epochs. The response patterns were largely consistent between the two time windows, but the modulation was relatively stronger for the later components of the population SC responses and V1 inputs in WT mice. In contrast, the modulation was rather stronger for the earlier response components in Scn2a$^{+/-}$ mice, or after chemogenetically blocking V1 inputs to SC in WT mice. No significant modulation was found in RGC axonal responses (Tukey-Kramer test for pairwise comparisons; see Supplementary Table 1 for details).

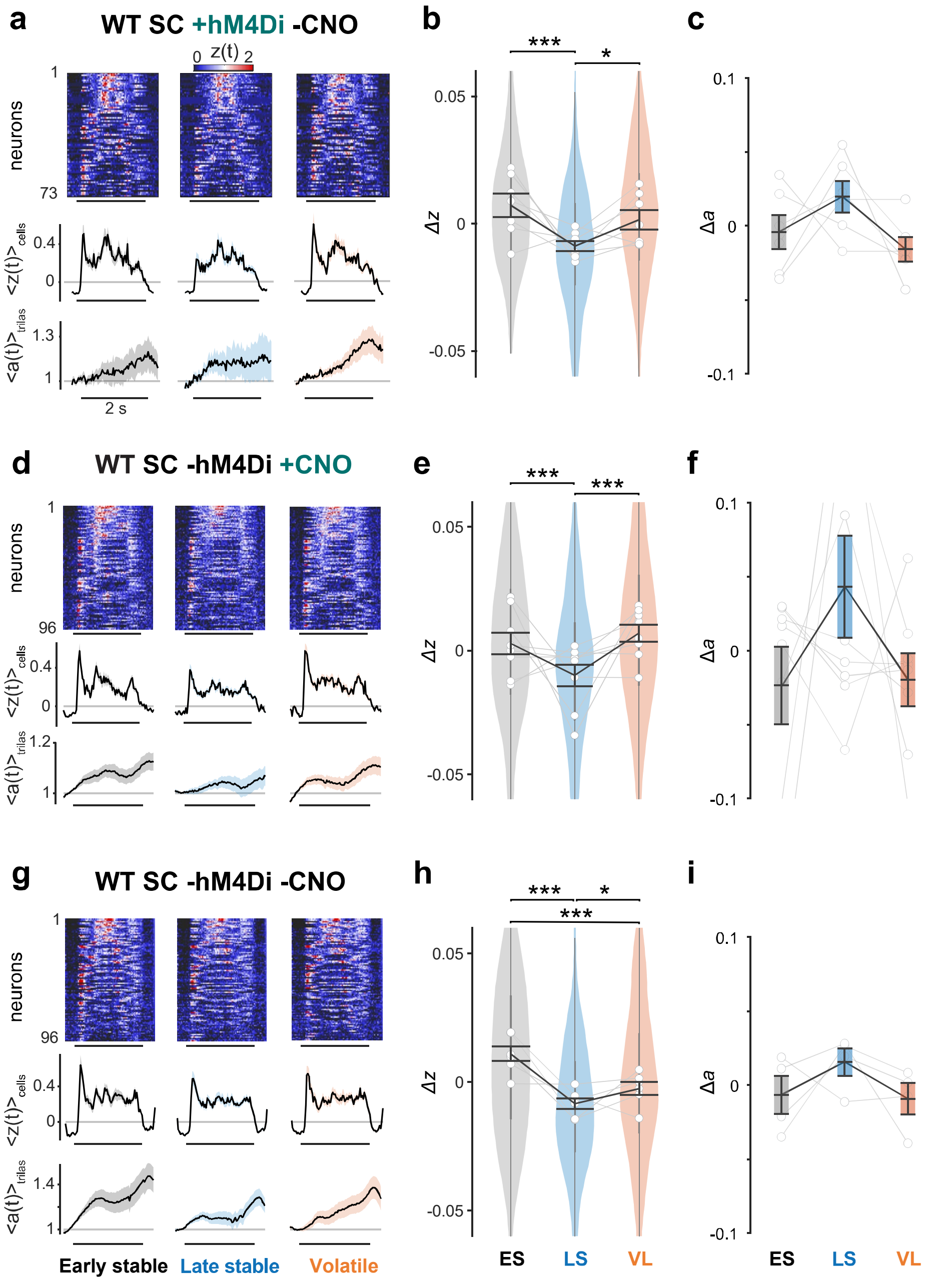

Supplementary Figure 5: hM4Di expression in V1 cells projecting to SC or CNO injection alone had no effects on context-dependent modulation of SC cells in WT mice.
a Aversive stimulus responses of simultaneously recorded SC neurons in a representative WT mouse expressing hM4Di receptors in V1 cells projecting to SC (top, heatmap $z(t)$ of 73 cells, sorted with $max[z(t)] > 1$; middle, population average $\langle z(t)\rangle_{\text{cells}}$; bottom, corresponding pupil dynamics $\langle a(t)\rangle_{\text{trials}}$; shown in the same format as Fig. 2d,g). **b** Distributions of the normalized SC responses across epochs (Δz) from WT animals expressing hM4Di in V1-to-SC projection (405 cells from 7 mice; shown in the same format as Fig. 2e,h). The data from the same animals after CNO systemic injection are shown in Fig. 5c,d. **c** Normalized change in pupil size ($\Delta a = \overline{a} - \langle \overline{a}\rangle_{\text{epochs}}$) from the same animals as in **b** (6 mice; thin line, individual animal; error bars, population average with s.e.m.). The data from the same animals after CNO systemic injection are shown in Fig. 6j. **d-f** Corresponding data for WT mice without hM4Di expression but with CNO injection (**c**, representative data from simultaneously recorded SC cells, 96 cells; **d**, population data for WT mice with CNO injection, 573 cells in total from 8 mice. **f**, population data for changes in pupil size as in **c**, 8 mice). **g-i** Corresponding data for a subset of mice shown in **d-f**, assessed without the systemic injection of CNO (**g**, representative data from simultaneously recorded SC cells, 96 cells; **h**, population data, 486 cells in total from 6 mice; **i**, population data for change in pupil size as in **c** (4 mice). This is a subset of data shown in **Fig. 2e**.

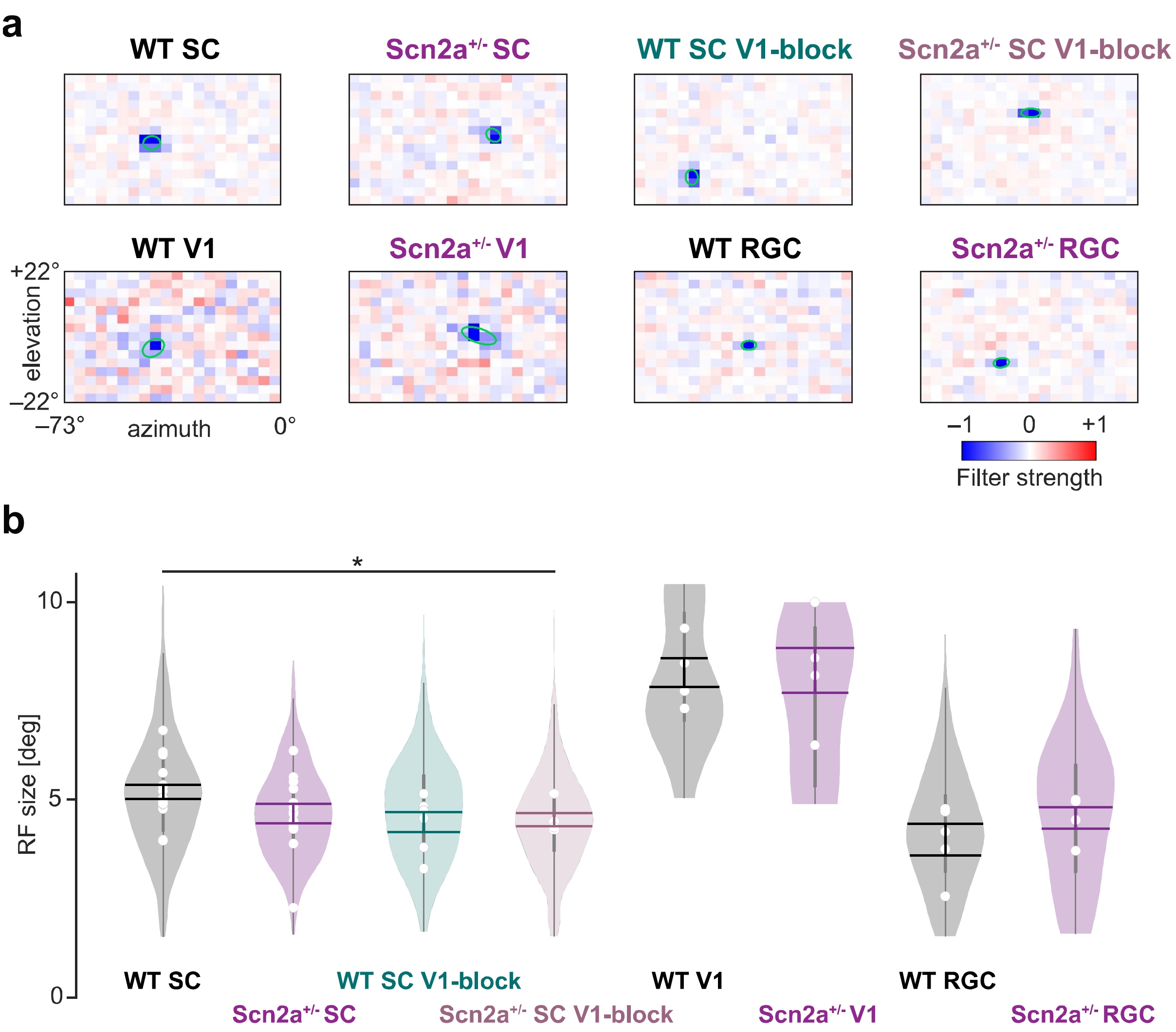

Supplementary Figure 6: The early visual systems of WT and Scn2a+/- mice have similar receptive field sizes.

a Representative spatial receptive field (RF, estimated by a statistical ensemble approach; see Methods for details) of SC cells (top; from left to right, WT, Scn2a+/-, WT with V1-block, and Scn2a+/- with V1-block), V1 cells projecting to SC (bottom left) and RGCs (bottom right), respectively. The green ellipse shows a contour of Two-dimensional Gaussian curve fit at 1 standard deviation. **b** Distribution of RF sizes. No substantial difference was found between genotypes (except for between SC cells in WT mice and SC cells in Scn2a+/- mice with V1-block): open circle, average for each mouse; vertical thick gray bar, interquartile range; horizontal lines, mean ± s.e.m.; *, $p<0.05$ with Tukey-Kramer test for pairwise comparisons. WT SC, 5.2±0.2; Scn2a+/- SC, 4.7±0.3; WT SC with V1-block, 4.4±0.3; Scn2a+/- SC with V1-block, 4.5±0.2; WT V1, 8.2±0.4; Scn2a+/- V1, 8.3±0.6; WT RGC, 4.0±0.4; Scn2a+/- RGC, 4.5±0.3 degrees.

Supplementary Table 1: FDR-adjusted p-values for contextual modulation analysis (Tukey-Kramer test). Texts in bold show those below a significance threshold (0.05).

genotype	chemogenetics	data type	stimulus type	p(ES-LS)	p(ES-VL)	p(LS-VL)	# cells	# animals	relevant figure #
WT	-	RGC	horizontal	1	0.65	0.057	304	5	S2d
WT	-	RGC	vertical	0.26	0.99	0.64	234	5	S2h
WT	-	RGC	omitted	0.9	0.99	1	138	5	S3e
WT	-	RGC	aversive<br>early<br>late	0.99<br>0.99<br>0.75	0.99<br>0.38<br>0.95	0.94<br>0.81<br>0.99	877	5	3c<br>S4a<br>S4b
WT	-	SC	horizontal	0.99	0.6	1	449	15	S2b
WT	-	SC	vertical	1	1	1	572	15	S2f
WT	-	SC	aversive<br>early<br>late	**0**<br>**4.2e-06**<br>**0**	0.27<br>0.99<br>0.79	**8.8e-17**<br>**0.04**<br>**3.2e-15**	1252	17	2e<br>S4a<br>S4b
WT	-	V1	horizontal	1	1	**0.02**	654	8	S2d
WT	-	V1	vertical	0.99	**5.6e-3**	**2.7e-4**	279	8	S2h
WT	-	V1	omitted	**0.02**	**1.8e-9**	0.3	249	8	S3e
WT	-	V1	aversive<br>early<br>late	**2.4e-5**<br>0.5<br>**2e-3**	1<br>0.99<br>0.99	**5.1e-3**<br>0.66<br>0.35	1376	8	4c<br>S4a<br>S4b
WT	-	pupil	horizontal	0.99	0.91	0.5	n/a	25	S2c
WT	-	pupil	vertical	0.065	0.76	0.98	n/a	25	S2g
WT	-	pupil	omitted	0.4	0.7	1	n/a	25	S3c
WT	-	pupil	aversive	0.5	0.7	**0.001**	n/a	25	6h
WT	hM4Di+CNO	SC	horizontal	1	1	1	168	6	S2b
WT	hM4Di+CNO	SC	vertical	0.99	**1e-4**	**0.032**	215	6	S2f
WT	hM4Di+CNO	SC	aversive<br>early<br>late	1<br>0.2<br>0.93	0.6<br>**3.3e-10**<br>0.4	0.98<br>0.13<br>0.99	423	7	5d<br>S4a<br>S4b
WT	hM4Di	SC	aversive	**2.9e-6**	0.99	**0.02**	405	7	S5b
WT	CNO<br>saline	SC	aversive	**3.9e-9**<br>**0**	0.055<br>**5.6e-5**	**0**<br>**0.01**	573<br>486	8<br>6	S5e<br>S5h
WT	hM4Di+CNO	pupil	horizontal	0.9	0.99	0.35	n/a	8	S2c
WT	hM4Di+CNO	pupil	vertical	1	1	1	n/a	8	S2g
WT	hM4Di+CNO	pupil	omitted	0.98	1	0.99	n/a	8	S3c
WT	hM4Di+CNO	pupil	aversive	0.15	1	0.9	n/a	8	6j
$Scn2a^{+/-}$	-	RGC	horizontal	**3.3e-4**	0.8	0.6	335	5	S2d
$Scn2a^{+/-}$	-	RGC	vertical	0.12	0.73	0.99	254	5	S2h
$Scn2a^{+/-}$	-	RGC	omitted	0.2	1	0.99	444	5	S3e
$Scn2a^{+/-}$	-	RGC	aversive<br>early<br>late	0.99<br>0.99<br>0.99	0.75<br>0.15<br>0.99	0.99<br>0.96<br>0.99	1208	5	3f<br>S4a<br>S4b
$Scn2a^{+/-}$	-	SC	horizontal	0.6	1	**4.8e-7**	424	15	S2b
$Scn2a^{+/-}$	-	SC	vertical	1	0.6	0.7	464	15	S2f
$Scn2a^{+/-}$	-	SC	aversive<br>early<br>late	0.29<br>0.17<br>0.99	**0**<br>**0**<br>**1.7e-11**	**0**<br>**0**<br>**3.2e-8**	1201	16	2h<br>S4a<br>S4b
$Scn2a^{+/-}$	-	V1	horizontal	**3.3e-4**	0.8	0.6	257	7	S2d
$Scn2a^{+/-}$	-	V1	vertical	0.12	0.73	0.99	252	7	S2h
$Scn2a^{+/-}$	-	V1	omitted	0.2	1	0.99	494	7	S3e
$Scn2a^{+/-}$	-	V1	aversive<br>early<br>late	0.99<br>**0.0048**<br>0.99	0.99<br>0.93<br>0.19	0.85<br>0.99<br>0.99	1254	7	4f<br>S4a<br>S4b
$Scn2a^{+/-}$	-	pupil	horizontal	0.99	0.99	1	n/a	24	S2c
$Scn2a^{+/-}$	-	pupil	vertical	0.99	1	0.99	n/a	24	S2g
$Scn2a^{+/-}$	-	pupil	omitted	0.5	1	0.9	n/a	24	S3c
$Scn2a^{+/-}$	-	pupil	aversive	0.4	1	0.7	n/a	24	6i
$Scn2a^{+/-}$	hM4Di+CNO	SC	horizontal	1	0.98	0.9	208	6	S2b
$Scn2a^{+/-}$	hM4Di+CNO	SC	vertical	0.99	0.65	**0.0088**	192	6	S2f
$Scn2a^{+/-}$	hM4Di+CNO	SC	aversive<br>early<br>late	0.99<br>0.09<br>0.99	0.99<br>0.98<br>0.99	1<br>0.99<br>0.99	550	7	5f<br>S4a<br>S4b
$Scn2a^{+/-}$	hM4Di+CNO	pupil	horizontal	0.85	0.74	1	n/a	7	S2c
$Scn2a^{+/-}$	hM4Di+CNO	pupil	vertical	1	1	1	n/a	7	S2g
$Scn2a^{+/-}$	hM4Di+CNO	pupil	omitted	1	1	0.99	n/a	7	S3c
$Scn2a^{+/-}$	hM4Di+CNO	pupil	aversive	1	1	1	n/a	7	6k

Supplementary Table 2: **Correlation analysis results between neuronal and HGF model parameter dynamics, related to Figure 7.** The number of cells significantly correlated with each of the 13 model parameters (in columns) is shown in each experimental group (in rows): top, positive correlation; middle, negative correlation; bottom below dotted line, FDR-adjusted p-value from a shuffling test against a polarity bias: blue, significantly more negatively correlated cells; red, significantly more positively correlated cells.

genotype	Area (Total *n*) / q-value	Lr1	Lr2	Lr3	Pp1	Pp2	Pp3	Bv2	Bv3	Un1	Un2	Un3	Pe2	Pe3	Subtotal: *n*(R>>0) *n*(R<<0)	% total corr. cells
WT	SC	13	0	37	14	11	29	10	31	29	10	2	1	0	187	28%
	(1080)	7	1	1	9	8	3	40	9	4	6	26	0	0	114	
	15 mice	0.6	0.6	7e-4	0.6	0.6	0.02	0.6	0.2	0.03	0.6	0.01	0.6	0.6		
	SC V1-block	5	0	0	11	12	2	1	7	2	5	1	1	0	48	23%
	(343)	2	0	0	12	9	1	1	4	1	1	2	1	0	33	
	6 mice	1	1	1	1	1	1	1	1	1	1	1	1	1		
	V1	51	0	10	61	77	19	7	0	27	46	0	1	2	301	38%
	(1254)	19	3	2	47	45	5	8	1	10	17	21	1	0	179	
	8 mice	0.1	0.6	0.6	0.6	0.4	0.3	0.6	0.6	0.4	0.2	0.01	0.6	0.6		
	RGC	0	0	3	0	0	0	0	1	0	0	0	0	0	4	0.6%
	(998)	0	0	0	0	0	0	0	0	0	0	2	0	0	2	
	5 mice	1	1	1	1	1	1	1	1	1	1	1	1	1		
$Scn2a^{+/-}$	SC	14	0	17	42	37	9	10	7	14	14	6	0	1	171	24%
	(1060)	0	0	13	14	8	11	13	20	3	1	4	0	0	87	
	15 mice	0.07	0.6	0.6	0.2	0.07	0.6	0.6	0.6	0.5	0.2	0.7	0.7	0.7		
	SC V1-block	11	1	1	15	14	4	1	3	4	8	2	1	0	72	34%
	(450)	3	2	7	20	18	2	7	16	3	3	8	0	0	82	
	6 imce	1	1	1	1	1	1	1	1	1	1	1	1	1		
	V1	0	0	7	2	2	2	0	2	1	0	0	5	3	24	5%
	(1376)	5	0	0	1	1	0	6	0	1	6	31	0	0	51	
	7 mice	0.9	0.9	0.9	0.9	0.9	0.9	0.9	0.9	0.9	0.9	0.003	0.9	0.9		
	RGC	9	0	10	0	0	3	0	3	0	8	0	0	1	34	6%
	(1208)	8	3	3	0	0	2	0	0	3	3	18	1	0	41	
	5 mice	0.9	0.9	0.9	0.9	0.9	0.9	0.9	0.9	0.9	0.9	0.1	0.9	0.9		
